# Supplementary material for: Patient Experience from a Pilot Study Implementing Software-Based Post-COVID Case Management in GP Practices—A Qualitative Process Evaluation
Source: Healthcare (Basel). 2025 Jul 15;13(14):1701. doi: 10.3390/healthcare13141701 (PMC12294771; doi:10.3390/healthcare13141701)
Supplement: Supplementary file 1 [file healthcare-13-01701-s001.zip › Figure_S2_Interview_Guide.pdf]

Figure S2 Interview Guide

"PostCovidCare - Pilot Study on the Implementation of a Post-COVID Case-Management with a browser-based symptom diary in the GP practice"

Guidelines for interviewing patients

Stage directions for the interviewers

- Interviewer has the FEWEST speaking time, open questions
- Inquiring formulations:
  - Be still - don't give up too soon
  - What did you find interesting/annoying/pleasant etc. about it?
  - I don't really understand it yet, can you explain it again?
  - Can you remember a situation?
  - What does this have to do with xxx?
  - What exactly do you mean by that?
  - Could you explain a situation that...

Procedure

- Documentation Interview Excel spreadsheet "Interviews_PCC-Pilot_Sociodemographics", if the patient has given their consent and has been infor-med. This includes age, gender, rural or urban place of residence, years of connec-tion to the current GP practice, duration of post-COVID symptoms in months.
- Pseudonym assignment - paper folder
- Mark audio file with pseudonym
- Sociodemographics available?
- Clarification available?
- Save interview as X folder

Before recording

Access

Hello, my name is X, I am (position) and I will be conducting the interview with you today.

Before we start, I would like to repeat the most important points about the process and you will have the opportunity to ask questions.

- I will now explain the process again: Phone on loudspeaker, digital recording device next to it, audio file is uploaded to a protected server in HD, to which only the study team has access. Pseudonymization, transcription, deletion of the audio file after transcription. If something is published, then always in such a way that neither the person nor the prac-tice can be identified.
- Voluntary - can be canceled at any time.
- We need about 30 minutes. It's more of a conversation, so just tell us. We are inte-rested in your opinion, your point of view. If you would like to take a break or stop the interview, please let us know.
- Do you have any questions before we start the recording?

I would start the recording now, okay? ◎ ON

Thank you for taking part in the interview. As already discussed, this is about your ex-perience of participating in the PostCovidCare pilot study. Part of the study involved regular appointments with your GP and documentation of your symptoms.

Did you use a paper-based symptom diary (or the Televital)?

Before we start with the specific questions, I have a few socio-demographic questions:

- Are you over or under 40 years old?
- Would you describe your place of residence as urban or rural?

A. Reach (how do I reach the target group - patients?)

Tell us: How did you come to take part in the study?

- How did you find out about participating?
- What motivated you to take part in the study?
- To what extent did your ability to work play a role in your decision to participate?

B. Implementation (how the intervention is used)

In the next section, I would like to find out from you how the appointments at the practice actually went. If you think back - there were 3 appointments at your GP practice, how did the appointments go?

- To what extent did you have to do any preparatory work?

First of all, you should go through a few questionnaires with them and measurements should be taken.

- What happened in your case?
- Who has taken on this task?
- What did you find annoying? What was successful?

This should be followed by a personal meeting in which, among other things, the next steps should be dis-cussed.

- What happened in your case?
- Who has taken on this task?
- What exactly was discussed?
- To what extent has an action plan been drawn up for the next steps?
- What information material did you receive at the appointments? To what extent were you able to use them?
- What did you find annoying? What was successful?

Then there was the symptom diary/televital, where you could enter your symptoms.

- To what extent did you use the Televital?
- How often are they used? In which situations?
- What did you find annoying? What was successful?

- To what extent were there differences between the first and subsequent dates?
- What happened after the last appointment?

C. Adoption (expectations, obstacles, disappointments)

Now that we've talked about how the appointments went. How would you rate them overall?

- What expectations did you have beforehand?
- Have these expectations been fulfilled?

D. Efficacy (positive and negative effects of the intervention)

Now I would like to know from you: What was it like for you to attend the appointments? How did you ex-perience them?

- What has changed as a result of participation compared to previous care?

E. Maintenance (How can I ensure the long-term use of the tool?)

We are now faced with the question of how to continue with PostCovidCare. You have gained experience and taken part in the appointments.

- What should be retained in any case?
- What did you miss? What would you have liked?
- What advice would you give us? What advice would you give the practice staff?
- What advice would you give other patients when they ask whether they should take part in the program?

F. Conclusion

We have now reached the end of the interview and have talked about many aspects.

- What else would you like to add?

Thank you very much for the interview and your time! Following our conversation, I will interview more patients and also the practice staff of the participating practices. The interviews will then be written up, the re-sults evaluated and then published. If you wish, I can send you the work after publication.
