# Supplementary material for: Patient Experience from a Pilot Study Implementing Software-Based Post-COVID Case Management in GP Practices—A Qualitative Process Evaluation
Source: Healthcare (Basel). 2025 Jul 15;13(14):1701. doi: 10.3390/healthcare13141701 (PMC12294771; doi:10.3390/healthcare13141701)
Supplement: Supplementary file 1 [file healthcare-13-01701-s001.zip › Table_S1_Code_Book.pdf]

Table S1 - Codebook

Main code	Subcode	Definition	Anchor example
Carrying out tests and questionnaire s	Questionnaires	Describes the experience of patients in receiving questionnaires, where they were completed and how they were supported.	"I had already received the documents beforehand, just like now. I have to say, at the second appointment I think I just had to fill out the questionnaire again, I would say now, I also have to say that my memory is just not the best anymore due to corona. The final appointment was just now and that was basically also the questionnaire, I had to pick it up beforehand and then bring it with me to the final interview." (Interview PAT_1, item 22)
	Physical and cognitive examinations	Describes the examinations and surveys of the state of health in the GP practice.	"Well, the physical examinations were blood pressure, oxygen saturation, things like that, and the other examinations were cognitive abilities, physical resilience." (Interview PAT_2, item 36)
	Perception of the patients	Addresses patients' perceptions during physical and cognitive examinations and questionnaires.	"In the beginning, well, it was very exhausting. Answering all the questions and everything and, yes, then sitting outside and filling out the questionnaire, then going back inside, then back at the PC with, er, answering questions and so on. That, that was exhausting, I have to be honest." (Interview PAT_7, pos. 34)
Doctor's consultation	Question about state of health	Describes the enquiry about the state of health in a personal interview.	"I mean that was another file where she asked me about it, where she entered it herself and yes, of course, how I'm doing and all the situations, how I'm coping." (Interview PAT_4, item 28)
	Discussion of the questionnaires	Includes patients' experiences as to whether completed questionnaires were discussed again during the interview or whether further information was entered on the PC in this context.	"He went through what I had prepared, i.e. the questionnaires that I had filled out, and we discussed them again together [...]" (Interview PAT_2, item 54)
	Completed symptom diary	Discusses the patient's experience of whether or to what extent the completed symptom diary was taken into account during the consultation. This	"And I always brought it to him. He looked through it and then we talked about it again briefly." (Interview PAT_10, pos. 46)

Main code	Subcode	Definition	Anchor example
		also includes statements on whether it was handed in at the GP practice.	
	Creation of an action plan	Describes the patient's experience of discussing and prescribing the necessary or existing therapies and specialist consultations based on the symptoms or other recommendations and the procedure for further care.	"And we just discussed that, and so basically we went through the initial interview again, all the symptoms I have and what the study suggests for my specific symptoms." (Interview PAT_10, pos. 34)
	Revision of the action plan	Includes the patient's experiences of the renewed discussion and, if necessary, correction of the action plan drawn up. As well as the experiences of discussing therapies utilised or specialist visits attended during the monitoring appointments.	"[...] he just wanted to know whether I had the feeling that it would bring something [...]" (Interview PAT_1, pos. 36)
	Receive information material?	Describes the experience of receiving information material from the GP or VERAH.	"I actually only received these post-COVID flyers from you. [...] But apart from that, I didn't get any information." (Interview PAT_6, pos. 66)
	Use of the information materials	The extent to which the interviewees were able to use the information material they received and how this helped them.	"Yes, I printed it out. At home and put it in a folder so that I always have it to hand and can do these exercises bit by bit." (Interview PAT_7, item 74)
	Inclusion of the PAT	Includes patient reflection on involvement in decision-making and explanation of processes.	"And then we sort of discussed it together and decided what I, what we were going to tackle." (Interview PAT_10, pos. 24)
	Desire for feedback	Describes the desire for information on the results and categorisation of the data collected.	"But I really would have liked to talk about it afterwards. So that's the point that I criticise, that perhaps it could have been tackled." (Interview PAT_8, item 112)
	Taken seriously	Includes the feeling of being taken seriously in a doctor's consultation.	"[...] and [he] was very interested in improving my general condition." (Interview_PAT_2, pos. 72)
	Time taken	Includes the evaluation of the time aspect of the doctor's consultation.	"The doctor took an incredible amount of time [...]" (Interview PAT_2, pos. 28)

Main code	Subcode	Definition	Anchor example
	Evaluation of doctor's consultation	Describes the extent to which the contents of the interview seemed useful or not useful to the interviewees. It also addresses the discussion of the symptom diary, but not statements on the usefulness of the symptom diary in general.	"Well, the fact that it was actually just the same thing that I had already filled out in the diary, I could have just handed it in." (Interview_PAT_3, item 134)
Symptom diary	Technical problems	Describes the interviewees' experiences of technical problems with the online symptom diary.	"The diary, on the internet, that didn't work at all." (Interview_PAT_5, pos. 70)
	Use of the symptom diary	Discusses experiences regarding the frequency of filling out the symptom diary (in which specific situations, how often and whether it is used at all).	"So I did it every three days at most, every three to four days, if there was a day when I didn't do so much, then I didn't have to enter anything or if there was something going on, then I realised, so I always did something every three days at most. (Interview PAT_5, item 72)
	Information on the symptom diary	Describes the respondents' missing information on: Availability of the online version of the symptom diary, regularity of completion and procedure after submission of completed forms.	"Several times I also used the, how should I put it, there was some address, I think the email address, that you could contact if it wasn't working, I did that too. And I never heard back and the doctor's surgery told me that they would be informed when it worked again at some point. But I never received this information, so I don't know whether the homepage worked at some point. I didn't try it again for myself" (Interview PAT_1, item 2)
	Become aware of your own health status	Addresses the extent to which respondents became aware of their own state of health as a result of completing the questionnaire and what this triggered in them.	"It was really, really cool to actually document it, so to speak. So, of course I know what's not going well for me and what restrictions I've had since corona. But it's quite something to list it all again. I don't know, it's difficult to put into words. But it's a different feeling than when it's just hovering somewhere in the back of your mind." (Interview PAT_10, pos. 52)
	Valuation	Describes the interviewees' views on the usefulness and suitability of the symptom diary as well as additional wishes in this regard. In addition, the code addresses the extent to which the scope of the symptom diary matched their needs or requirements	"[...] with the diary that was good, it helped me a lot, [...]" (Interview PAT_5, pos. 108)

Main code	Subcode	Definition	Anchor example
		and their opinion on whether they would find the symptom diary online or paper-based better.	
Effects	Use of other healthcare services after prescription	Describes mentions of consultations with medical specialists and the use of therapies as part of the intervention as well as the evaluation of effectiveness.	"So the fact that I got this breathing therapy helped me anyway." (Interview_PAT_10, pos. 58)
	Implementation of other proposals	Addresses the experiences of implementing suggestions for lifestyle changes that respondents received as part of the intervention.	"I go to the gym three times a week, if I can manage it somehow. And I do low-level endurance training, because when it comes to the heart you always say go over the limit, but when it comes to the lungs you don't go over the limit. And with the equipment in the gym, I can check this well, if I were to go jogging, it wouldn't be so easy. Then I would have to do it with a pulse (watch), which only works moderately for me." (Interview PAT_10, pos. 74)
	Change in care of post-COVID disease	Addresses whether and, if so, to what extent the care of post-COVID disease has changed as a result of participation in the study or describes what the care looks like after the end of the intervention.	"No, just the system with the lung doctor again, with the asthma spray, which I then take, but nothing else." (Interview PAT_4, item 72)
Patients' overall experience of the intervention	Organisation of appointments	Describes the processes in the GP practice, including waiting times and appointment schedules as well as appointment arrangements.	"I didn't have to wait in the waiting room or anything like that. That actually worked, I went in there and was channelled through straight away." (Interview PAT_9, pos. 26)
	Differentiation of dates	Includes the extent to which the appointments differ from the patient's perspective.	"The first was very extensive and the second and third were less extensive." (Interview PAT_2, pos. 106)
	Time and intensity	Addresses the respondents' assessment of the time spent and the extent to which the practice staff actively engaged with the patient during the appointment.	"But it's somehow become a bit tighter. Simply through the study. Because we kept talking about it." (Interview PAT_6, pos. 126)

Main code	Subcode	Definition	Anchor example
	Division of tasks between general practitioners and VERAH	Describes the interviewees' experiences of which tasks were performed by the VERAH and which by the GPs.	"So the check-up, as I said, was done by the head of the practice [GP], so this physical examination and the blood count was done by the lady who always takes blood samples and the other things [...]" (Interview PAT_1, pos. 24)
	Total valuation	Includes the respondents' assessment of appointments at the GP practice as a whole.	"Yes, the whole thing was just good. It worked well. Yes, there was a bit of a problem with the PC, but that's just technology. But all in all, it actually went well. In terms of the dates, in terms of the support, everything actually went well." (Interview PAT_7, pos. 130)
The future of intervention	More support - not further specified	Addresses the patients' desire for more support, but this is not explained in depth.	"Yes, that you might also get other support somehow, [...]" (Interview PAT_3, pos. 18)
	More information	Describes the interviewees' desire for more information material, e.g. with regard to technical terms, the disease and treatment options.	"So as I said, if the study is continued or reissued, I think it would be important to simply give people a bit of information material as a tool." (Interview PAT_1, item 76)
	More concrete support offers	Includes respondents' desire for support in starting a therapy and exercises to do at home.	"[...] I don't know, maybe mindfulness exercises for someone who has long Covid, memory games or something to train memory, I think I wouldn't find something like that bad." (Interview PAT_1, pos. 78)
	Further development of the intervention	Describes the patient's wishes for the future of the intervention.	"I don't know if you could divide the study into different parts, like me, it's incredibly hard on my lungs, on my endurance, on the oxygen in my blood. I wonder if you couldn't form groups so that you could go into the individual cases more specifically, for example one person has problems with their lungs, another has problems with their joints, another has problems with their heart and so on." (Interview PAT_4, pos. 94)
	Recommendations to patients	Addresses the extent to which and, if applicable, under what circumstances the respondents would recommend the study to other patients with post-	"As the effort involved was not that great for me personally, I would definitely do it, because you might get occupational therapy, which you may not have had before, and simply get a

Main code	Subcode	Definition	Anchor example
		COVID disease and where they see the advantages and disadvantages of participating.	thorough check-up, at least at my doctor's surgery." (Interview PAT_1, item 70)
